# Development of novel human in vitro vascularized adipose tissue model with functional macrophages

**DOI:** 10.1007/s10616-020-00407-6

**Published:** 2020-06-10

**Authors:** Outi Huttala, Jertta-Riina Sarkanen, Marika Mannerström, Tarja Toimela, Tuula Heinonen, Timo Ylikomi

**Affiliations:** 1 FICAM, Faculty of Medicine and Health Technology, University of Tampere, Tampere, Finland; 2 Cell Biology, Faculty of Medicine and Health Technology, University of Tampere, Tampere, Finland

**Keywords:** hASC, Adipose tissue derived endothelial cells, Interferon gamma, Adipogenesis, In vitro model development, Adipose tissue derived macrophages

## Abstract

Inflammation has been proven significant factor in development of type 2 diabetes. So far, most of the adipose tissue related research has been performed in animals, mainly rodent models. The relevance of translation of animal results to humans is questionable. However, in vitro model with relevant human cell source, such as human adipose tissue stromal cells (hASC), can be developed and should be utilized for human adipose tissue research. We developed in vitro models of human adipose tissue utilizing hASC, endothelial cells and monocytes/macrophages. By isolating endothelial cells and macrophages from same adipose tissue as hASC, we were able to provide method for constructing personalized models of adipose tissue. With these models, we studied the effect of macrophages on adipogenesis and protein secretion, with and without vasculature. The models were analyzed for immunocytochemical markers, cell number, triglyceride accumulation and protein secretion. We found that lipid accumulation was greater in adipocytes in the presence of macrophages. Interferon gamma increased this difference between adipocyte culture and Adipocyte–Macrophage co-culture. Protein secretion was affected more by macrophages when vasculature was not present compared to the mild effect when vasculature was present. The vascularized adipose model with macrophages is valuable tool for human adipose tissue research, especially for the personalized medicine approaches; for choosing the right treatments and for studying rare medical conditions.

## Introduction

During the recent decades, the incidence of obesity has increased dramatically throughout the world. As the prevalence of obesity increases the amount of related diseases increases. Obesity is associated with type 2 diabetes as well as cancer, sleep apnea, asthma, degenerative joint disease, hypertension, renal failure, stroke, and cardiovascular disease (Switzer et al. 2013, van Baak 2013). Hence, adipose tissue research, particularly studies of disturbances in normal tissue function, is ever more important. Adipose tissue contains heterogeneous cell population and has multiple functions including energy storage, endocrine functions and maintenance of metabolic homeostasis. Adipose tissue is composed of adipose stem cells, mature adipocytes, immune cells, fibroblasts, endothelial cells. Adipocytes are the main cell type in adipose tissue. Due to their large size, they occupy 90% of the volume of adipose tissue (Kanneganti and Dixit 2012).

Inflammation of adipose tissue has been suspected to be key reason behind many of the complications such as type 2 diabetes mellitus (the loss of insulin sensitivity). In adipose tissue, insulin resistance manifests as impaired glucose disposal and enhanced triglyceride lipolysis, resulting in hyperinsulinemia, hyperglycemia, and hyperlipidemia (Shulman 2000). Obesity leads to enlargement of adipocytes and in addition, promotes macrophage accumulation in adipose tissue (Gil et al. 2007; Murano et al. 2008). Obese people have been shown to have a continuous low-grade inflammation in their adipose tissue (Gil et al. 2007). The first evidence of link between inflammation, obesity and insulin resistance was the finding that increased expression of tumor necrosis factor α (TNFα), an inflammatory cytokine, promoted insulin resistance via serine phosphorylation of IRS1 (Hotamisligil et al. 1993, 1996).

Interferon gamma (IFNγ) produced by macrophages is one of the markers involved in adipose tissue inflammation. Obese IFNγ-deficient animals have been shown to have significantly reduced expression of inflammatory genes TNFα and monocyte chemoattractant protein-1, decreased inflammatory cell accumulation, and better glucose tolerance than control animals (Rocha et al. 2008). In addition, obese IFNγ-knockouts, have demonstrated improved insulin sensitivity and decreased adipocyte size (O’Rourke et al. 2012). Adipose stem cells have been shown to inhibit the Th1 phenotype and subsequently decrease IFNγ and TNFα production but also increase the secretion of IFNγ production from Th17 cells (Eljaafari et al. 2015).

Along with inflammation, theories on the reasons leading to insulin resistance include the impaired adipogenesis (Okuno et al. 2018). The process of stem cells maturing into adipocytes is called adipogenesis. Adipogenesis would lead to formation of new adipocytes and hence relieve the stress of over expanding adipocytes. When the adipocytes over-expand the stress signals get activated (Haczeyni et al. 2018). This in turn influences the function of mitochondria and leads to the state of inflammation (Codoner-Franch et al. 2011, Patti and Corvera 2010). It is still not quite clear which event in this cascade finally causes the insulin resistant stage in adipocytes. Information of adipogenesis in the presence of inflammation and studies on the role of these numerous factors known to take part in the formation of insulin resistance would lead to the possibility of new treatment methods for obesity related diseases. Also, the role of different cells in the development of insulin resistance is still largely unknown. Personalized in vitro models with cells from only one patient could also result in even better insights of the development of these diseases.

Relevant and reliable adipose tissue research requires suitable tools to achieve high quality results. Although the natural heterogeneity of adipose tissue is achieved when using animal models, the species-to-species variation in translating these results is too great flaw to dismiss (Seok et al. 2013). Murine models have been extensively used to identify and test drug candidates prior to clinical trials (European Commission 2010; US Department of Health and Human Services Food and Drug Administration, Center for Drug Evaluation and Research Guidance for Industry 2006; Woodcock and Woosley 2008). However, only few of these candidates have been successful in the human trials (Hackam and Redelmeier 2006; van der Worp et al. 2010). The success rates for those trials and animal experiments, where the studied disease involves inflammation, seem to be especially low (Mitka 2011; Seok et al. 2013). As an alternative, in vitro models have been developed. However, most in vitro models developed so far contain animal cells including 3T3-L1 adipocytes but lack all other components of adipose tissue (Ruiz-Ojeda et al. 2016). These may shed light on some aspects of adipose tissue function but are not optimal for studying human adipose tissue biology. Hence, the need for better models is evident for the research of human adipose tissue and its complications, especially inflamed adipose tissue.

Human adipose tissue is a vast and easy source of cells, which are relevant due to their human origin. Human adipose tissue stromal cells (hASC), obtained from adipose tissue, are widely used, due to their well-established isolation and culturing methods, plasticity, easiness to obtain and lack of ethical concerns. One great advantage for them is the ability to produce them in reproducible manner and in ways that allow clinical use (Patrikoski et al. 2013). Consequently, hASC have been utilized in many different in vitro tissue models including vasculature/angiogenesis models (Huttala et al. 2015; Merfeld-Clauss et al. 2010), adipose tissue models (Huttala et al. 2016, 2018; Volz et al. 2018) and in cartilage and bone tissue engineering (Ojansivu et al. 2015; Vuornos et al. 2016).

Here we present adipose tissue models, which combine in vitro human adipocytes (differentiated from hASC), vascularization (composed of human umbilical vein endothelial cells (HUVEC) and hASC) and human primary monocytes differentiated to macrophages. Others have developed similar human hASC-immune cell co-culture models (Eljaafari et al. 2015; Kongsuphol et al. 2019; Liu et al. 2019) but have not contained endothelial cells. Also, no other endothelial cell containing adipose tissue model including immune cells were found in literature which would utilize natural adipogenesis induction (Sarkanen et al. 2012a, b). The use of natural adipogenesis induction (Adipose Tissue Extract, ATE) is also beneficial for the vascular development compared to the commonly used chemical adipogenesis cocktail (Kang et al. 2009; Sheu et al. 2006).

Our goal was to develop versatile models which allow studying the effects of inflammation markers like IFNγ on adipocytes, immune cells and vasculature. Also, the aim was that these models allow the studies of the interaction between adipocytes, vasculature (endothelial cells) and immune cells. In these developed models we specifically study the effect of macrophages by comparing each culture set-up to a same set-up lacking the macrophages. Finally, for the personalized medicine purposes, the developed method would allow the use of single donor adipose tissue sample (isolation the endothelial cells and macrophages from adipose tissue along with hASC). This type of protocol provides a relevant model for the needs of personalized medicine.

## Materials and methods

### Ethical considerations

This in vitro study conforms to the ethical principles outlined in the Declaration of Helsinki. The human adipose tissue samples and human umbilical cords were received with written informed consent from Tampere University Hospital, Tampere, Finland. The use of adipose tissue derived cells and human umbilical cord endothelial cells (HUVEC) were approved by The Regional Ethics Committee of Tampere University Hospital’s Responsibility Area, Tampere, Finland with permit numbers of R15161 and R15033, respectively. The blood samples from which monocytes were isolated, were ordered from Finnish Red Cross Blood service, and used with their permit number 35/2014.

### Isolation and treatment of hASC, HUVEC and monocytes

#### Isolation and culture hASC and HUVEC

Isolation of hASC and HUVEC is depicted in Sarkanen et al. (2012a, b). Before cryopreservation, the cells were screened for mycoplasma utilizing MycoAlert^®^ Detection Kit (Lonza Group LTD, Basel Switzerland) and were mycoplasma free. The used batches of hASC were proven to express CD73, CD90 and CD105 (BD) as described previously (Huttala et al. 2015). Same cell batch of hASC (adipose tissue obtained from one patient) was utilized throughout this study. The adipose tissue sample was subcutaneous fat from abdomen of obese female who was not known to suffer from any metabolic disorder. Also, same batch of HUVEC (umbilical cord from another patient) was used throughout this study when HUVEC were utilized. hASC were propagated in hASC medium (Table 1). HUVEC were propagated in EGM-2 Endothelial Cell Growth Medium-2 (Lonza). In the models, hASC were used at passage 2 and HUVEC at passage 4.

**Table 1:** Media utilized in the study, their content and manufacturer

Medium name	Components	Manufacturer
hASC medium	10% Human Serum 2 mM l-Glutamine in Dulbecco’s Modified Eagle’s Medium Nutrient Mixture F-12 (DMEM/F12)	Lonza Gibco (Carlsbad, CA, USA) Gibco
HUVEC medium	EGM-2 Endothelial Cell Growth Medium-2	Lonza
Serum free medium (SFM)	ITS (1.15 µM insulin, 6.65 µg/ml Transferrin, 6.65 ng/ml seleniuous acid) 2.56 mM l-glutamine 1% Bovine serum albumin 2.8 mM Sodium Pyruvate 50 U/ml Penicillin/50 µg/ml Streptomycin 0.1 nM 3,3′,5-Triiodo-L-thyronine sodium salt 10 ng/ml Vascular endothelial growth factor A 1 ng/ml fibroblast growth factor 2 100 µg/ml Ascorbic acid 50 ng/ml heparin 0.2 µg/ml hydrocortisone in DMEM/F12	BD Biosciences (NJ, USA) Gibco Biosera (Boussens, France) Gibco Gibco Sigma Aldrich (MO, USA) R&D Systems (Abingdon, UK) R&D Systems Sigma-Aldrich Sigma Aldrich Sigma Aldrich Gibco
Monocyte medium	50 U/ml Penicillin/Streptomycin in RPMI-1640	Gibco ATCC
ATE medium	1700 µg/ml of ATE 10% HS 2 mM l-Glutamine 50 U/ml Penicillin/Streptomycin in DMEM/F12	Own production, see Chapter 4.4 Lonza Gibco Gibco Gibco
Monocyte-to-macrophage medium	10% inactivated Human serum in RPMI-1640	Lonza ATCC

#### Isolation of monocytes from human blood

Monocytes were isolated from blood utilizing Ficoll Paque Plus (GE Health care). Blood sample diluted with PBS was added on top of Ficol and these tubes were centrifuged for 40 min at 400 G without brakes. Buffy coat was collected, and PBS washes were performed three times, first centrifugation at 350 *g* for 10 min, second at 200 *g* for 10 min and third at 350 *g* for 10 min. This pellet, containing mononuclear cells from blood, was resuspended to PBS and suspension was filtered through 30 µm filter (BD), centrifuged at 300 g for 10 min and supernatant was removed. To separate T cells from this cell suspension, human Pan T cell isolation kit (Miltenyi Biotec, Bergisch Gladbach, Germany) and MACS magnetic separator midiMACS (Miltenyi Biotec) were utilized according to the manufacturer instructions. Cells were suspended to buffer and Pan T cell biotin-antibody cocktail was added and incubated at 4 °C for 10 min. Pan T cell Microbead cocktail was then added and incubated at 4 °C for 15 min. After wash and centrifugation at 300 *g* for 10 min, the supernatant was removed, and cells were suspended to the buffer and added into the MACS LS column (Miltenyi Biotec). The unlabeled flow-through is the T cell population, which was not utilized in the study, and cells attached to the column include monocytes. Monocytes were cultured in monocyte medium (Table 1). Cells were cryopreserved in RPMI-1640 (ATCC) supplemented with 10% DMSO (Sigma), 10% human serum (Lonza type AB filtered) and 50 U/ml Penicillin and streptomycin (Gibco).

#### Differentiation of monocytes to macrophages

Monocytes, isolated from blood as well as commercial monocytes i.e. CD14 + Monocytes from Peripheral blood, single donor (PromoCell, C-12909), were utilized. After thawing, the cells were cultured in a 25 cm^2^ culture bottle in monocyte-to-macrophage medium (Table 1) to differentiate the monocytes towards macrophages. Human serum in the medium differentiates monocytes into macrophages (Andreesen et al. 1983; Musson 1983). For the first 24 h after thawing, the cells were allowed to recover. Then fresh medium was changed. After 3 days, some of monocytes had attached to the bottom and differentiated to macrophages, whereas some of monocytes were growing in suspension. Every 4–5 days, half of the medium was changed, and the morphology of the cells was monitored by microscope. After 10 days, cells growing were subcultured with ratio of 1:2 into 25 cm^2^ culture bottles. After culturing the cells for 24 days, the macrophages were differentiated and ready to be used in the construction of the in vitro models. Maturity was tested with methods presented in paragraph “Analysis of isolated and differentiated macrophages”. Detachment of macrophages was done by Macrophage detachment solution DXF (PromoCell, C-41330) and scraping with a cell scraper.

### Isolation of cells from adipose tissue for the personalized model

CD11b positive cells and endothelial cells (CD31 positive) were isolated from the same adipose tissue sample as hASC.

#### Isolation of CD11b positive cells from adipose tissue

CD11b selection was used to obtain immune cells (mainly monocytes/macrophages) from adipose tissue. The adipose tissue was cut into small pieces and incubated in 0.15% collagenase I (Gibco) for 12 h. This was then centrifuged at 600 *g* for 10 min followed by the removal of the supernatant. To remove red blood cells by bursting them, the pellet was incubated in sterile water for 2 min. DMEM/F12 (Gibco) supplemented with 1% l-glutamine (Gibco), 10% Human serum (Lonza) and 50 U/ml Penicillin and streptomycin (Gibco) was added to dilute the water and this was centrifuged at 600 *g* for 10 min which resulted in cell pellet (the tissue was processed similarly up to this step also for endothelial cell isolation). The cell pellet was resuspended and filtered through 100 µm mesh. The CD11b positive cells were isolated utilizing mouse/human CD11b Microbeads kit (Miltenyi Biotec) and MACS magnetic separator miniMACS (Miltenyi Biotec) according to manufacturer’s instructions. Cells in the adipose tissue derived cell suspension were counted and divided 1 x 10^7^ cells per tube followed by incubation with CD11b antibody for 10 min at + 4 °C and wash with MACS running buffer. Cell suspension was then centrifuged at 300 *g* for 5 min, supernatant was removed, and fresh running buffer added. This suspension was then run through a MACS column with pre separation filter (Miltenyi Biotec). Column was washed three times before collecting the cells captured into the column. Cells were cultured in Monocyte medium (Table 1) prior to analysis of their functionality.

#### Isolation of endothelial cells from adipose tissue

Pluribead anti-human CD31 S-bead kit (PluriSelect, Leipzig, Germany) was utilized for positive selection of endothelial cells from adipose tissue. The adipose tissue was treated as described in paragraph “Isolation of CD11b positive cells from adipose tissue” until cell pellet was reached. The pellet was resuspended in the buffer of the Pluribead kit and the cell number was adjusted to be max 5 x 10^6^ target cells/ml and this suspension was filtered through provided filter. CD31 S-Pluribeads were then mixed with the cell suspension and incubated at room temperature (RT) for 30 min on rotation on MACSmix™ Tube Rotator (Miltenyi Biotec). This suspension was then filtered through equilibrated strainers. The strainer was then transferred on top of sterile 50 ml tube, washed and detachment buffer was added and incubated for 10 min at RT. Cells were released from the strainer and strainer was washed with HUVEC medium. This suspension was then centrifuged 10 min at 300 x g without brake. The pellet was suspended into EGM-2 Endothelial Cell Growth Medium-2 medium (Lonza) and transferred into 25 cm^2^ culture bottle.

### Preparation of adipose tissue extract

In order to obtain adipose tissue extract (ATE) (Lopez et al. 2016, 2018; Sarkanen et al. 2012a, b), human adipose tissue specimens were mechanically cut into small pieces and incubated in DMEM/F12 (Gibco) in +37 °C for 1–24 h. After the incubation, the liquid was collected, centrifuged and sterile filtered and stored in − 70 °C until use. Protein concentrations of ATE was determined utilizing Pierce BCA Protein Assay Kit (Thermo Scientific, Waltham, MA) according to manufacturer’s instructions using bovine serum albumin as a standard. Results were measured after 30 min incubation at 37 °C at 562 nm with Varioskan Flash Multimode Reader (Thermo Scientific).

### Analysis of isolated and differentiated macrophages and endothelial cells

#### LDL uptake by CD11b positive cells and endothelial cells

Low density lipoprotein 488 (LDL-488, Molecular probes, Eugene, Oregon, USA) at 10 µg/ml concentration was added on cells and incubated for 4 h at 37 °C. After incubation, cells were washed and analyzed by microscope and imaged with automated imaging system Cell-IQ (Chipman Technologies, Tampere, Finland).

#### Test of engulfment function of macrophages

To ensure the successful differentiation of macrophages, fluorescein stained cell debris was fed to the macrophages. This debris was produced by staining hASC with cell membrane label mini-67 PKH67 Green fluorescent cell linker kit (Sigma) according to manufacturer’s instructions. Cells were washed with PBS (Gibco), detached from the culture bottle, centrifuged 400 x g for 5 min followed by removal of supernatant. The pellet was suspended into residual supernatant. Diluent was added on cells followed by addition of PKH67 at concentration of 4 µM. This was incubated for 5 min in RT and mixed once during this time. Equal amount of hASC medium was added to the tube and centrifuged 400 *g* for 10 min. Washing was performed two times in a fresh tube using hASC medium. This stained cell suspension was then frozen and thawed for four times. The breakdown of cells was confirmed by microscopy. Cell debris was stored at − 20 °C until used. Stained cell debris was added on macrophages and the engulfment of these particles was visualized and recorded for 5 h using automated imaging system, Cell-IQ (Chipman technologies).

#### Hematoxylin–eosin staining of macrophages

Hematoxylin–eosin staining was performed by pipetting cell suspension containing macrophages on a microscopy glass and embedding them with Tissue-Tek^®^ O.C.T. Compound (Miles inc. Elkhart, IN, USA). Absolut alcohol, 94% ethanol, 70% ethanol and distilled water were added on the glass in sequence each for 1 min. Mayer hematoxylin was added for 10 min and this was rinsed with water and then with distilled water. 1% Eosin was added for 2 min followed by 96% ethanol, absolute ethanol and xylene. Cells were imaged with Nikon Eclipse TS100 inverted fluorescence microscope (Nikon, Tokyo, Japan) and Nikon digital sight DS-U2 –camera (Nikon).

### In vitro cell models developed in the study

#### Adipocyte–macrophage Model

The development of adipose tissue depicting cell models was started by combining the macrophages and hASC i.e. creating Adipocyte–Macrophage model (AMM). In this model adipogenesis was induced with ATE and the angiogenesis induction medium (serum free medium, SFM, Table 1) was utilized to obtain more mature adipocytes as published earlier (Huttala et al. 2018). SFM was also used to confirm that it is suitable medium for sustaining macrophages. No cytokines were added except those found in ATE.

hASC were plated on 48 well plate at density of 20,000 cells/cm^2^ in hASC medium (Table 1). Detachment of hASC was done by Tryple Express (Gibco). Monocytes isolated from blood were seeded on top of hASC at density of 7500 cells/cm^2^ in the Monocyte medium (Table 1). One day after the plating, the medium was changed to ATE medium (Table 1). On day 4, medium was changed to SFM (Table 1) which was replenished every third day. Analysis were performed on day 13. To analyze the triglyceride accumulation Adipored (Lonza) reagent was utilized and for viability measurements, WST-1 (Roche, Basel, Switzerland) reagent was utilized.

Control (AMM without macrophages) was cultured in exactly same manner as AMM but lacked the macrophages. The medium compositions and protocol summary can be seen in Table 1 and 2, respectively.

**Table 2:** Construction of the Adipocyte–Macrophage model (AMM) and its control

Model/control	Day 0	Days 1	Day4	Day 7	Day 9	Day 13
AMM without macrophages	Plating of hASC	ATE medium	SFM	SFM	SFM	Analysis
AMM	Plating of hASC and monocytes	ATE medium	SFM	SFM	SFM	

#### Adipocyte–macrophage model with IFNγ and vascularized adipose tissue model with macrophages with IFNγ

We further optimized the AMM to allow studies of effects of cytokines. The optimization was done keeping in mind the addition of endothelial cells in the later stage. Hence the protocol was developed so that this protocol would also allow the addition of endothelial cells without changes to the protocol. IFNγ was utilized as example cytokine.

The following protocol was developed to construct Adipocyte–Macrophage model with IFNγ (AMMI). On day 0, hASC plated on 48 well plate at density of 20,000 cells/cm^2^ in hASC medium (Table 1). On day 1, the medium was changed to ATE medium. ATE has been previously shown to induce natural adipogenesis (Sarkanen et al. 2012a). On day 8, the medium was changed to serum free medium (Table 1). On day 11, the medium was replenished, and macrophages were added at a density of 34,000 cells/cm^2^ in serum free medium. ATE was added in the wells in final protein concentration of 387.1 µg/ml.

On day 12, 20 ng/ml of IFNγ (R&D Systems) was added in wells and on day 13, IFNγ was added to concentration of 60 ng/ml. On day 21, the whole medium from the wells was collected, frozen and stored in − 80 °C until analysis by ELISA. To analyze the triglyceride accumulation Adipored (Lonza) reagent was utilized. Control was cultured in exactly same manner as the AMMI except lacked macrophages. The media used and protocol outlines can be seen in Tables 1 and 3, respectively.

**Table 3:** Construction of the Adipocyte–Macrophage model with IFNγ (AMMI) and vascularized adipose tissue model with IFNγ (VATMI). SFM = serum free medium

Model	Day 0	Days 1–7	Day 7	Days 8–10	Day 11	Day 12	Day 13	Day 21
AMMI	Plating of hASC	ATE medium		SFM	Plating of macrophages, SFM supplemented with ATE	20 ng/ml of IFNγ	Addition of 60 ng/ml IFNγ	Analysis
AMMI without macrophages	Plating of hASC	ATE medium		SFM	SFM with ATE	20 ng/ml of IFNγ	Addition of 60 ng/ml IFNγ	
VATMI	Plating of hASC	ATE medium	Plating of HUVEC	SFM	Plating of macrophages, SFM supplemented with ATE	20 ng/ml of IFNγ	Addition of 60 ng/ml IFNγ	
VATMI without macrophages	Plating of hASC		Plating of HUVEC	SFM	SFM supplemented with ATE	20 ng/ml of IFNγ	Addition of 60 ng/ml IFNγ	

To analyze the cytokine content of the medium samples collected from AMMI, Human obesity ELISA Strips (Signosis, Silicon valley, CA, USA) containing TNFα, insulin-like growth factor 1 (IGF), resistin, interleukin 6 (IL-6), plasminogen activator inhibitor 1 (PAI), transforming growth factor β (TGFβ), adiponectin and leptin were used according to manufacturer’s instructions. Standards, controls and samples were incubated on the plate for 1 h at RT with shaking. After washes, the biotin-labelled antibody mixture was incubated for 1 h at RT with shaking followed by washing steps. Streptavidin-HRP conjugate was added and incubated for 45 min at RT with shaking. After washes, substrate was added and incubated for 20 min at which point the stop solution was added. The optical density was measured at 450 nm with plate reader Varioskan Flash (Thermo Scientific).

To construct the Vascularized Adipose Tissue model with Macrophages with IFNγ (VATMI) similar protocol to AMMI was developed, except for the additions. On day 7, HUVEC were plated in EGM-2 medium (Lonza) at density of 4000 cells/cm^2^. The cells were added into the medium in which hASC had grown for the week. Control samples were cultured in the same manner as VATMI, except the macrophages were not added into the control wells. The media used and protocol outlines can be seen in Tables 1 and 3, respectively. To analyze the cytokine content of the medium samples collected from VATMI, Human inflammation ELISA strips (Signosis) containing TNFα, granulocyte-colony stimulating factor (G-CSF), granulocyte-macrophage colony-stimulating factor (GM-CSF), interleukin 1a (IL-1a) and 8 (IL-8), interferon gamma-induced protein 10 (IP-10 also known as CXCL10) and rantes were used according to manufacturer’s instructions and as described above.

### Analysis of models

#### Viability and triglyceride accumulation

The relative number of living cells was analyzed by WST-1 (Roche) with 1-h incubation. Absorbance was measured at 450 nm with Varioskan flash multimode reader (Thermo Fischer Scientific).

Triglyceride accumulation to the cell cultures was measured with Adipored assay reagent (Lonza) with 10 min incubation at RT after the WST-1 analysis. Fluorescence was measured with Varioskan flash multimode reader (Thermo Fischer Scientific), with excitation at 485 nm and emission at 572 nm. The Adipored values were normalized with WST-1 values to obtain relative amount of triglycerides per cell.

#### Immunocytochemical staining

To visualize the vascular-like network formation and macrophages in the co-culture immunocytochemical stainings were performed. The immunocytochemical staining was performed as described earlier (Huttala et al. 2015) except for the fixative used here was 4% paraformalaldehyde at RT for 20 min. After fixation, the cells were permeabilized with 0.5% Triton-X100 (MP Biochemicals, Ohio, USA) and non-specific binding sites were blocked with 10% BSA (Roche). Primary antibody dilution in 1% BSA in PBS was applied on the cells. Primary antibodies were α Anti-von Willebrant factor IgG (produced in rabbit, Sigma), CD11b and CD68 (both from BD). Secondary antibodies were also applied in 1% BSA in PBS solution. Secondary antibodies used were TRITC-labeled goat polyclonal antibody anti-rabbit IgG (Sigma), FITC-labeled goat polyclonal antibody anti-mouse IgG (Sigma), DAB Peroxidase (HRP) Substrate Kit, (Vector labs) and V450 (BD). After immunocytochemical staining the vascular-like network was photographed with Nikon Eclipse TS100 inverted fluorescence microscope (Nikon) and Nikon digital sight DS-U2 –camera (Nikon). Images were further processed with NIS Elements (Nikon) and Adobe Photoshop CS3-software (Adobe Systems Incorporated, San Jose, CA, United States).

### Statistical analysis

All results were plotted and statistical analyzes were performed with GraphPadPrism (GraphPad Software Inc., California, USA). Results are depicted as mean ± standard deviation. Triglyceride accumulation comparisons (n = 6 in all) analyzed with Mann–Whitney test. For ELISA (n = 2) comparisons were performed with Two-way ANOVA with Sidak’s multiple comparisons test. Differences were considered significant when *p < 0.05, **p < 0.01 and ***p < 0.001.

## Results

### Isolated cells are functional

**Fig. 1:**
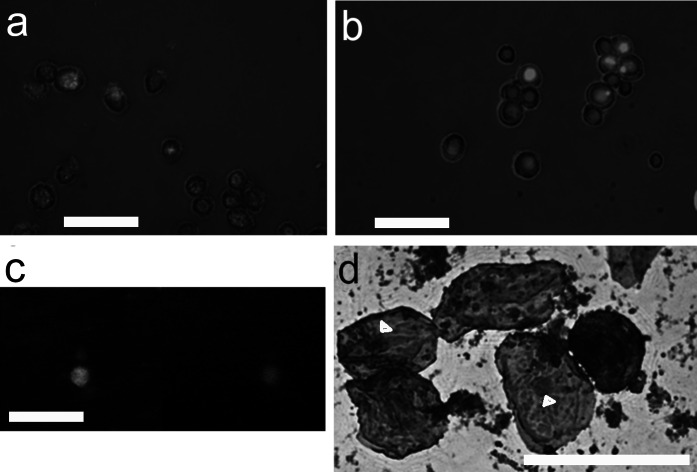
Identity and functionality of macrophages. Macrophages engulfed dead cell parts (shown in green), imaged with Cell-IQ (Chipman technologies). In image **a** in the first time point, the well of cell culture plate is still full of small particles seen as fuzzy green background. **b** At 5 h time point, the background is clean, because macrophages have engulfed the dead cell debris. **c** Double positive macrophage (CD68-FITC (green) and CD11b-V450 (blue)). **d** Hematoxylin–eosin stained macrophages isolated from adipose tissue. Nuclei of the macrophages marked with arrowhead. **c** and **d** obtained with Nikon Eclipse TS100 inverted fluorescence microscope and digital sight DS-U2 –camera. Scale bar 50 µm

As one of the goals was to be able to construct the model from one lipid sample, we tested the isolation methods for isolation of macrophages and endothelial from adipose tissue. When one patient model is not needed, the monocytes are isolated from blood and differentiated to macrophages. Also, endothelial cells are routinely obtained from umbilical cord (HUVEC). Macrophages and endothelial cells from all sources were tested for their correct phenotype and functional features. The isolated monocytes, which were differentiated towards macrophages, engulfed the cell debris they were given (Fig. 1). The uptake of LDL was also seen in both macrophages and endothelial cells which shows they are functioning normally. In addition, the staining of the macrophages proved them positive for CD11b and CD68 (Fig. 1).

### Presence of macrophages affects the lipid accumulation in adipocytes

**Fig. 2:**
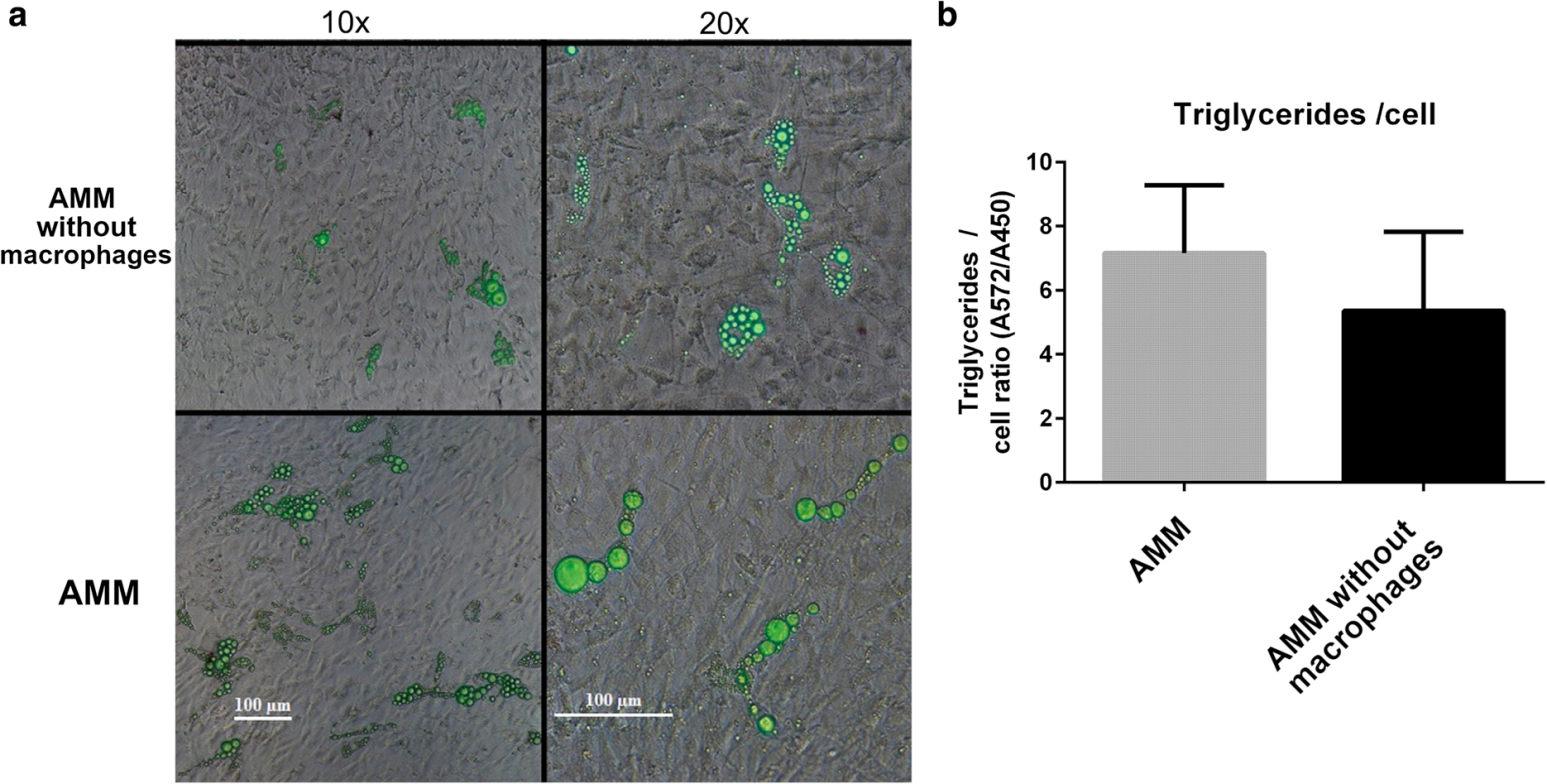
Adipocyte–Macrophage model (AMM). **a** Phase contrast image overlaid with fluorescence image of the model with and without macrophages. Triglycerides stained with Adipored, seen in green. The presence of macrophages seems to lead to larger lipid accumulation in adipocytes. Scale bar in both columns 100 µm. **b** Triglyceride accumulation per cell calculated from AdipoRed (total amount of triglycerides) and WST-1 (relative cell number) results. Images obtained with Nikon Eclipse TS100 inverted fluorescence microscope and digital sight DS-U2 –camera

The development of adipose tissue depicting cell models was started by combining the macrophages and hASC i.e. creating Adipocyte–Macrophage model (AMM). In this model adipogenesis was induced with ATE, a natural adipogenesis inducer. The impact of macrophages on lipid accumulation was studied in AMM by comparing it with AMM without macrophages. Figure 2a shows the morphology of the cell models. The interaction of adipocytes and macrophages leads to larger lipid vesicles in adipocytes (Fig. 2a). This difference was also seen in the analysis of amount of triglycerides per cell (cell number was relative total cell number of the model determined by WST-1) however, the difference was not significant (Fig. 2b). The larger lipid vesicles in adipocytes could indicate more mature state i.e. further and better differentiation, of the adipocytes in presence of macrophages.

### Adipocyte–Macrophage model with IFNγ

**Fig. 3:**
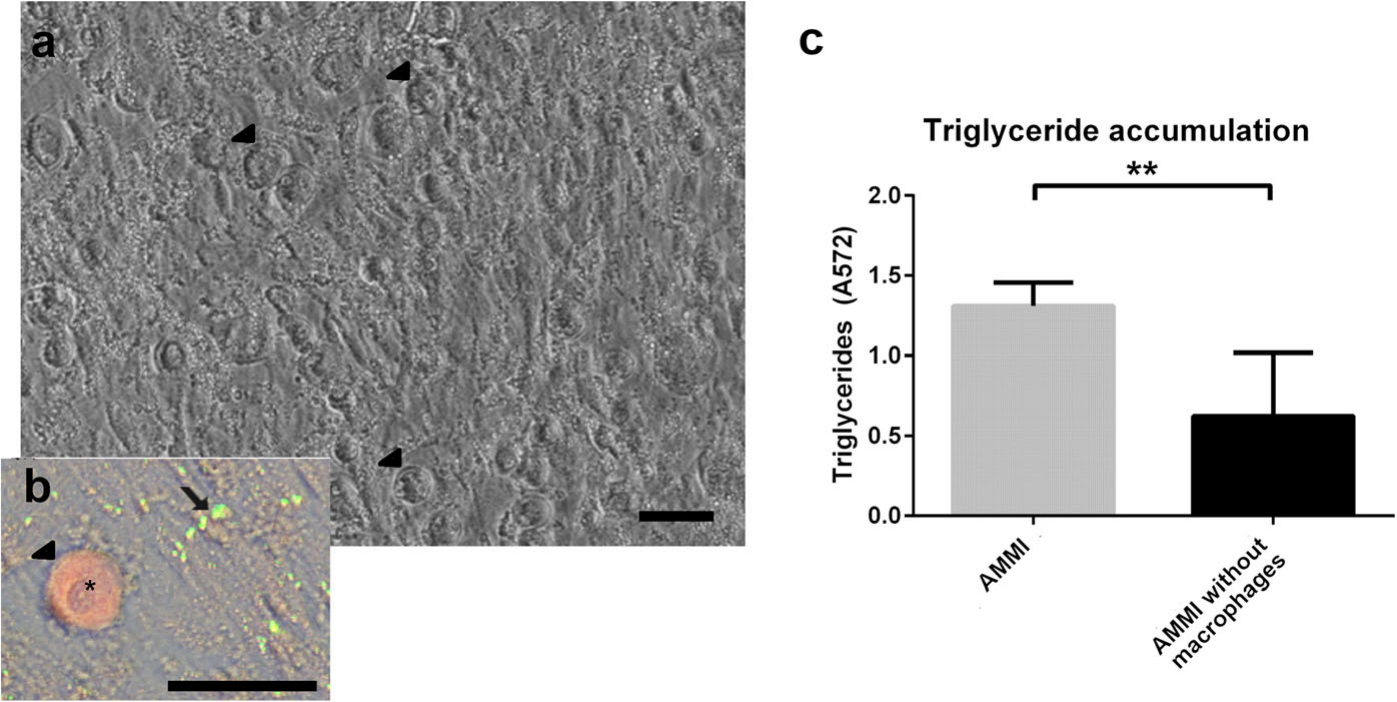
Adipocyte–Macrophage model with IFNγ (AMMI). Image **a** Phase contrast image of adipocytes and macrophages. Image **b** Close-up of macrophage in the culture: phase contrast image combined with AdipoRed (green) stained adipocytes and macrophage stained with CD11b (red). Macrophages marked with arrow heads and lipids stained with adipored marked with arrow. Macrophage nucleus can be seen in the close-up (asterisk). Obtained with Nikon Eclipse TS100 inverted fluorescence microscope and digital sight DS-U2 –camera. Scale bar 50 µm. **c** Triglyceride accumulation significantly differs between cultures with and without macrophages. Results depicted as mean ± SD, **p < 0.01

The Adipocyte–Macrophage model was further optimized to allow studies of cell–cell interactions together with studies of the effect of IFNγ (now called AMMI). This modification also allows other cytokines to be studied in the future. In this co-culture setting, the differentiation of hASC to adipocytes was studied by analyzing the lipid accumulation with or without the presence of macrophages. Macrophages attached on top of the hASC, which were differentiated into adipocytes (Fig. 3a). The immunostaining image (Fig. 3b) shows macrophage stained with CD11b in red and triglycerides inside the adipocytes in green (stained with AdipoRed). The triglyceride accumulation in adipocytes was significantly increased in the presence of macrophages and IFNγ (Fig. 3b). IFNγ has not changed the total body weight in animal studies to (Rocha et al. 2008) which could indicate the role of macrophages in increasing the lipid accumulation in presence of IFNγ.

**Fig. 4:**
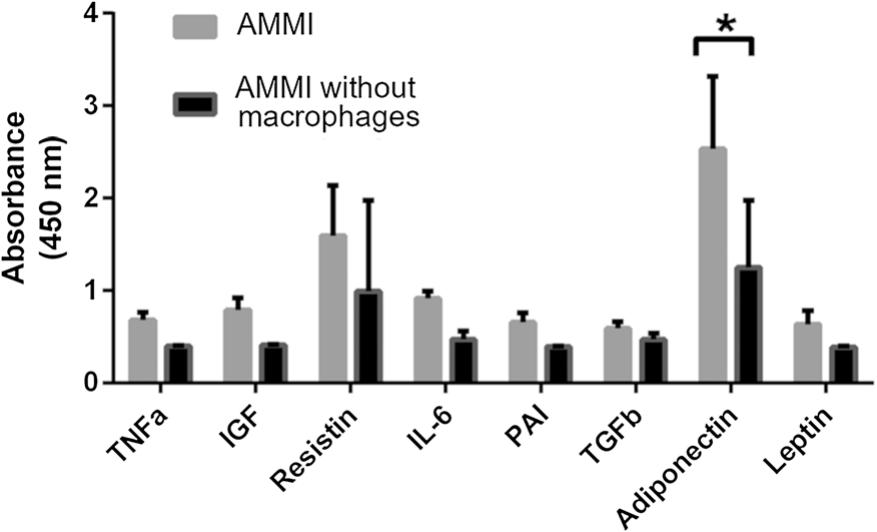
Cytokine expression in Adipocyte–Macrophage model with IFNγ (AMMI). Amounts of TNFα, IGF, Resistin, IL-6, PAI, TGFβ, Adiponectin and leptin were determined. Adiponectin shows significant increase in the presence of macrophages. Results depicted as mean ± SD, *p < 0.05

Secretion of proteins in AMMI was analyzed from the medium samples collected on day 21 of the culture (Fig. 4). Studied proteins were TNFα, IGF, resistin, IL-6, PAI, TGFβ, adiponectin and leptin as these are proteins found in adipose tissue and they are secreted mainly from adipocytes and immune cells (Makki et al. 2013). Addition of macrophages increased the secretion of adiponectin significantly. This was surprising as adiponectin favors insulin-sensitivity and IFNγ knockout rather than presence of IFNγ has been linked to modest improvements in insulin sensitivity and decreased adipocyte size (O’Rourke et al. 2012). Secretion of adipocyte and obesity related proteins IGF, PAI, and Leptin were increased in AMMI compared to AMMI without macrophages. Resistin secretion seemed to increase but due to the high variation of control treatment, this cannot be confirmed. Production of pro-inflammatory cytokines TNFα and IL-6 was also increased in AMMI. TGFβ secretion was not changed whether macrophages were present in the culture or not.

### Vascularized adipose tissue model with macrophages

**Fig. 5:**
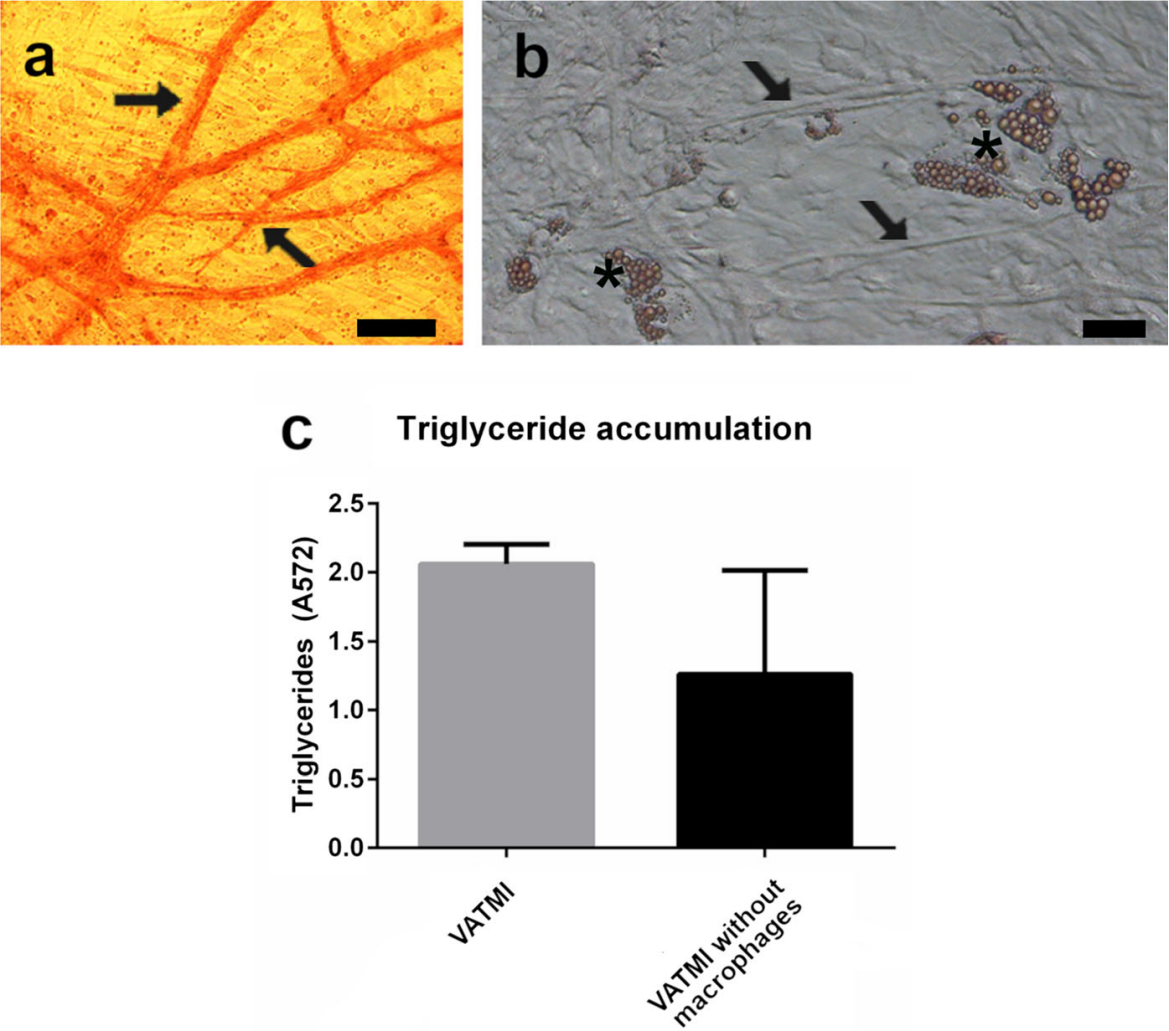
Characteristics of Vascularized Adipose Tissue model with Macrophages with IFNγ (VATMI). **a** Vasculature in VATMI stained with DAB. **b** Adipocytes and vessel structures in the phase contrast image of VATMI. Both obtained with Nikon Eclipse TS100 inverted fluorescence microscope and digital sight DS-U2 –camera. Vasculature marked with arrows and adipocytes with asterisks. Scale bar in both 100 µm. **c** Triglyceride accumulation differs between cultures with and without macrophages. Results depicted as mean ± SD

To study the effect of vascularization on lipid accumulation and protein secretion, the AMMI was further developed to form Vascularized adipose tissue model with macrophages and the same IFNγ additions as AMMI (VATMI). Vasculature is important part of adipose tissue and the diseases related to adipose tissue. Hence to properly depict the biology of adipose tissue all three systems need to be present; vasculature, adipocytes and immune cells. By combining the hASC, HUVEC and macrophages, we were able to achieve formation of vascular structures (Fig. 5a), differentiation of adipocytes, which collect triglycerides (Fig. 5b and c), and the activation of macrophages in the same culture. The addition of macrophages increased triglyceride accumulation in adipocytes also in the presence of vasculature. However, due to the high variation in the control this was not found significant.

**Fig. 6:**
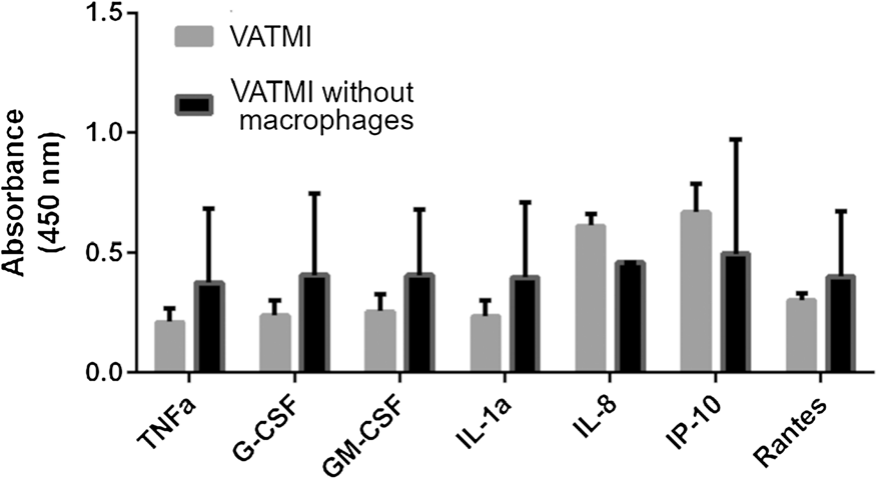
Cytokine expression in Vascularized Adipose Tissue model with Macrophages with IFNγ (VATMI). Increase is seen in IL-8 and IP-10 although none of the differences were significant. Results depicted as mean ± SD

Secretion of TNFα, G-CSF, GM-CSF, IL-1a, IL-8, IP-10 and rantes was studied from medium samples collected from VATMI on day 21. These selected proteins are found in adipose tissue and are produced by adipocytes, endothelial cells or immune cells (Makki et al. 2013; Nosalski and Guzik 2017). The focus was in studying the effect of macrophages/their absence in this model system and not in comparing AMMI and VATMI to each other. In addition to proteins secreted by adipocytes and immune cells, we looked at proteins produced by endothelial cells or production is affected by endothelia cells (Kawai et al. 1999; Mariotti et al. 2006) and which can influence the formation of vasculature (Bodnar et al. 2006; Li et al. 2003). Expressions of IL-8 and IP-10 (CXCL10), both inflammation related cytokines, increased in the presence of macrophages (Fig. 6), although the change was not significant. Overall, the presence of macrophages did not lead to change in expression or decreased the expression of proteins in VATMI however the large variations in results do not allow reliable interpretation.

## Discussion

We have previously developed in vitro vascular structures (Huttala et al. 2015) and vascularized adipose tissue model (Huttala et al. 2018), which were modified in the present study to construct vascularized adipose tissue models with macrophages. We were able to produce extensive vascular network, adipocyte differentiation and allow functionality of the macrophages in single co-culture set-up. Both Adipocyte–Macrophage model (consisting of hASC differentiated to adipocytes and macrophages) and Vascularized adipose tissue model with macrophages (consisting of hASC differentiated to adipocytes, macrophages and endothelial cells forming vasculature) are relevant tools for adipose tissue research.

In these in vitro models, the effect of macrophages on lipid accumulation as well as on inflammation marker expression was studied. In addition, to enable the use of this model in the context of personalized medicine, the proof-of–concept method of isolation of hASC, monocytes and microvascular endothelial cells from single patient was created. The results show that the model could be set-up by using cells from one patient as long as the adipose tissue sample is large enough. In addition, the presence of macrophages seemed to lead to larger lipid storages per adipocyte. However, the presence of vasculature seemed to attenuate the changes in protein secretion caused by the macrophages.

Due to the growing incidence of obesity, the adipose tissue research is more and more important in determining the mechanisms and finding novel treatment options for obesity and the related complications. In vitro models are known to be more relevant than animal models and therefore, the developed in vitro models should contain human cells. ASC are excellent cell type for relevant in vitro models. The definition of mesenchymal stem cells, including ASC, is that they must express CD105, CD73 and CD90 (Dominici et al. 2006). The characterization of the cells used in in vitro models is as important as the characterization of the model. We have previously characterized the hASC and HUVEC obtained with our optimized isolation protocol (Huttala et al. 2015). Hence, these cells have routine batch quality control and are not discussed further here. However, the isolated monocytes, the activated macrophages, adipose tissue derived macrophages and adipose tissue derived endothelial cells are characterized in this study. The monocytes, which were differentiated to macrophages, and the adipose tissue derived macrophages were all confirmed to be active and functional determined by their ability to clear out dead cell debris and uptake LDL. Due to the heterogeneity of macrophages (Gordon and Taylor 2005), the use of adipose tissue derived macrophages can be justified in in vitro models aiming to depict the inflammation in adipose tissue.

Although HUVEC are well studied and commonly used endothelial type in tissue engineering, the use of HUVEC has been criticized as they are derived from macro vessels. Hence, more relevant cell sources are needed, especially now that studies are showing more and more differences in characteristics between the endothelial cells isolated from different sources (Marcu et al. 2018). Here we isolated the endothelial cells from adipose tissue and confirmed the functionality with the uptake of LDL. These tissue specific human endothelial cells are highly relevant cell source for in vitro model development.

For needs of personalized medicine, the cells used in the in vitro models need to be obtained from single donor with as little additional disturbance to the patient as possible. Here we tested the methods and characterized the isolated cells and were able to isolate all the necessary cell types, macrophages, endothelial cells and hASC, from one patient adipose tissue sample. Hence, after further testing this in vitro model system of Vascularized adipose tissue model with macrophages could be utilized in the research of adipose tissue related diseases and in the screening of suitable treatment options for specific patients and patient groups.

The insulin metabolism contains many players, which also impact the formation of insulin resistance. The cell intrinsic mechanisms include mitochondrial dysfunction, oxidative and endoplasmic reticulum (ER) stress, and ectopic lipid deposition (Chawla et al. 2011). Alterations in circulating adipokines and fatty acids, and metabolic tissue inflammation are the dominant cell extrinsic pathways that modulate peripheral insulin action (Chawla et al. 2011). By utilizing in vitro models, these parts of processes and event cascades can be studied one by one to obtain new information on the roles of each event on the disease development such as type 2 diabetes and metabolic syndrome.

The successful construction of these models allowed the study of the impact of macrophages on hASC differentiation and adipocyte function. The presence of macrophages was found to induce larger lipid storage formation in adipocytes than adipocytes grown without macrophages had. Hence, interaction between macrophages and adipocytes/maturing adipocytes may be causing the formation of hypertrophic adipocytes in the obese individuals. This is in line with the previous studies which report that oxidative stress suppresses SREBF1, which is important in adipocyte differentiation (Okuno et al. 2018). Further studies would be needed to confirm the mechanisms by which this interaction leads to favoring the over expansion of adipocytes rather than adipogenesis of adipose stem cells.

The secretion of Adiponectin, IGF, PAI, and Leptin were increased in Adipocyte–Macrophage model with IFNγ (AMMI) compared to model lacking macrophages. Production of adiponectin reverses insulin resistance associated with both lipoatrophy and obesity (Yamauchi et al. 2001). Hence, the model still shows signs of insulin sensitivity, although we found lipid accumulation to increase in the presence of macrophages. Change in expression of adiponectin has been shown to be a marker of insulin sensitivity independent from body mass index (Matulewicz et al. 2017). The expression of adiponectin also indicates that hyperplasia instead of hypertrophy is depicted in the model. Hyperplasia has been characterized as increased production of adiponectin and down regulation of inflammatory adipokines, whereas in hypertrophy adiponectin is down regulated and inflammatory adipokine production increased (Torres-Leal et al. 2012). Hypertrophic adipocytes lose their ability to store fat and increase their lipolytic activity. This process allows an increase in free fatty acid plasma concentration that in turn may impair the function of non-adipose organs, a process called lipotoxicity (DeFronzo 2004). It has been shown that hypertrophic adipocytes impair insulin signaling in adipocytes and other organs through dysregulated secretion of adipocytokines, such as adiponectin (Maeda et al. 2002), TNFα (Uysal et al. 1997), IL-6 (Rotter et al. 2003), and resistin (Steppan et al. 2001). Consequently, the secretion of these proteins might indicate that the model could be modifiable to depict insulin resistant tissue. Due to the pro-inflammatory markers expressed by AMMI, it is likely that the model presents state of early obesity, still expressing adiponectin but also secreting the cytokines linked to hypertrophic adipose tissue.

The macrophage produced cytokines, TNFα and IL-6 (Coelho et al. 2013), were found to be secreted in the AMMI which indicates that the macrophages were active in the model. Although mature adipocytes secrete TNFα, macrophages in adipose tissue are the main source of the cytokines (Maury et al. 2009). In obese humans, serum TNFα concentration is elevated but can be reversed through weight loss (Dandona et al. 1998). In adipose tissue, TNFα reduces the expression and activities of PPAR-γ, lipoprotein lipase and Glut-4, and hence impacts adipocyte differentiation, glucose uptake and lipid storage (Imai et al. 2004; Zhang et al. 1996). TNFα inhibits PPARγ activity and consequently leads to suppression of adipocyte differentiation and several conditions including insulin resistance (Ye and Gimble 2011). The nuclear receptor PPARγ is a lipid sensor that promotes lipid accumulation through gene transcription. TNFα regulates PPARγ both at pre-translational and post-translational levels (Ye 2008).

From the other secreted proteins, PAI, IGF and leptin; PAI has been found to cause hypercoagulation (Singh et al. 2013). IGF has been shown to influence proliferation and differentiation of perivascular adipocytes as well as stimulate migration of endothelial cells and angiogenesis (Liu et al. 2015; Nakao-Hayashi et al. 1992; Shigematsu et al. 1999). Leptin is also involved in the proliferation and differentiation of preadipocytes but especially functions as hormone regulating energy balance suppressing food intake and thereby inducing weight loss (Halaas et al. 1995; Wagoner et al. 2006).

The ratio of macrophage subtypes has been shown to differ between lean and obese mice (Weisberg et al. 2003). Obese adipose tissue macrophages express general macrophage marker cd68 and CD11b, CD11c, CD86, CD32, CCR7 (Thomas and Apovian 2017).  As the isolated macrophages were positive for CD11b and CD68 they correlate with the previously reported adipose tissue macrophage marker expression. Obesity induces the accumulation of M1 macrophages to adipose tissue. M1 macrophages express IL-6, NOS, CCR2 (Lumeng et al. 2007) and TNFα, leading to a pro-inflammatory environment in adipose tissue (Ota 2013). Secretion of IL-6 was observed also in our model. Elevated plasma concentration of IL-6 is a marker for development of type 2 diabetes and for myocardial infarction (Pradhan et al. 2001; Ridker et al. 2000).

Both human serum (Andreesen et al. 1983; Musson 1983) and IFNγ have been used to differentiate monocytes into macrophages in vitro (Ralph et al. 1983). M1 macrophages are the subset that is activated by IFNγ (Murray and Wynn 2011). M1 macrophages express pro-inflammatory cytokines and inducible nitric-oxide synthase (Murray and Wynn 2011) and show high microbicidal activity and produce reactive oxygen species (Gordon and Taylor 2005).

In our results, the vasculature seemed to attenuate the effects caused by macrophages. However, the lipid accumulation was greater both with and without vasculature. The secretion of IL-8 and IP-10 (CXCL10) in VATMI indicates that the macrophages in the model are active. IP-10 has been shown to inhibit endothelial tubule formation (Bodnar et al. 2006). Although IP-10 was detected in VATMI it did not effect the formation of vascular network. It has been previously shown that expansion of adipose tissue vasculature counteracts obesity related inflammation and metabolic complications (Robciuc et al. 2016). This was seen in the decrease of TNFα, among other factors (Robciuc et al. 2016). Our results could indicate similar effect of vasculature on G-CSF, GM-CSF, IL-1a and Rantes expression but further studies would be needed to confirm such effect. Rodciuc et al. found that the expansion of vasculature in adipose tissue had no effect on body weight (Robciuc et al. 2016). Hence, the increased lipid accumulation seems to be influenced by the macrophages independently rather than from the amount of vasculature.

Mature adipocytes could be utilized the assay to ensure the maturity of the adipocytes. Earlier studies have shown that the in vitro differentiation of pre adipocytes might be resulting in less mature adipocytes (Volz et al. 2019). The maintenance and long-term culture of mature adipocytes has been shown to be successful by others (Harms et al. 2019; Louis et al. 2019). In the future this in vitro protocol could be carried out with mature adipocytes. However, at the moment our goal is to also look into the differentiation of preadipocytes in the presence of inflammatory components. This research of differentiation process and its interactions with immune cells and inflammation markers would allow research of type 2 diabetes and other complications seen in context with obesity. The maturity of the adipocytes obtained after this culture period will be further studied in the future.

Limitation of this study is that the results have been obtained with cells from one adipose tissue sample. At this method development stage there were a lot of variables with multiple cell types and their growth requirements to consider. Hence, by using one adipose tissue sample we were able to compare the different protocols. The results obtained at this stage of the model development can only show trends of the changes. Also, it would be interesting, but we cannot discuss on the effects of the source of the sample on the results obtained at this stage of the study. For such comparison we would need wider ethical permits to collect more patient data and the study needs to be conducted with multiple different patient samples to obtain reliable data on such comparison. As presented in this study, we now have a ready protocol for the future experiments and these will include comparison of results obtained from different patient sample types to see whether the biology of the cells in adipose tissue is different depending on the source, whether we can see the difference in in vitro and if we can determine the different mechanism behind these differences in obese vs. normal weight, healthy and diabetic patient.

Here the protocols are presented at proof-of-concept state and more extensive analyses will be conducted in the future. The protocols need further characterization to ensure their in vivo likeness. Monocytes/macrophages are not only immune cells present in the adipose tissue especially during the inflammation of the adipose tissue. Hence, the addition of other immune cell types should be investigated. The known and defined components and natural components used in the protocols make these kinds of changes easier than with the use of chemicals not found in vivo. For personalized medicine purposes, the amount of adipose tissue required for the model might be limiting factor in some cases.

## Conclusion

As the number of obese individuals continues to rise, adipose tissue research is ever more important. Adipose tissue research needs new relevant and reliable tools for various research areas related to the tissue: studies of disease development, roles of tissue components on the disease onset and progression, toxicological studies and development of new treatments. By increasing the knowledge of the mechanisms behind the diseases such as type 2 diabetes and metabolic syndrome, the development of new treatment options is easier.

Human adipose tissue is an excellent and ample source of cells suitable for use in in vitro models aiming to depict human tissues. hASC are especially suitable cell type for in vitro adipose tissue models. The human cell in vitro model developed here, Vascularized adipose tissue model with macrophages, is a valuable tool for investigating the role of inflammation cytokines, immune cells and vascularization in human obesity. As the presence of macrophages seems to be affecting the lipid accumulation characteristics of adipocytes, it should be studied further. The presence of vascularization seemed to attenuate the changes in protein secretions. This could lead to more relevant intervention methods for the obesity related diseases related to inflammation and vascular changes.

The in vitro model presented here is especially relevant for the personalized medicine approaches. Personalized in vitro models enable studies of individual disease variants. Hence, the in vitro models utilizing one patient material would be especially important for disease studies in the rare disease types. Further efforts are still needed to fully utilize these new research tools in research as well as in drug development and diagnostic applications.

## Abbreviations

AMM: Adipocyte–Macrophage Model
AMMI: Adipocyte–Macrophage Model with IFNγ
ATE: Adipose tissue extract
G-CSF: Granulocyte-colony stimulating factor
GM-CSF: Granulocyte–macrophage colony-stimulating factor
hASC: Human adipose stromal cells
HUVEC: Human umbilical vein endothelial cells
IFNγ: Interferon gamma
IGF: Insulin-like growth factor 1
IL-1a: Interleukin 1a
IL-6: Interleukin 6
IL-8: Interleukin 8
IP-10: Interferon gamma-induced protein 10/CXCL10
LDL: Low density lipoprotein
PAI: Plasminogen activator inhibitor 1
RT: Room temperature
SFM: Serum free medium
TGFβ: Transforming growth factor β
TNFα: Tumor necrosis factor α
VATMI: Vascularized Adipose Tissue model with Macrophages with IFNγ
